# Migrants With Schizophrenia in Forensic Psychiatric Hospitals Benefit From High-Intensity Second Language Programs

**DOI:** 10.3389/fpsyt.2021.711836

**Published:** 2021-08-11

**Authors:** Maximilian Lutz, Judith Streb, Larissa Titze, Michael Büsselmann, Nadja Riemat, Christian Prüter-Schwarte, Manuela Dudeck

**Affiliations:** ^1^Department of Forensic Psychiatry and Psychotherapy, Ulm University, Ulm, Germany; ^2^Vitos Clinic for Forensic Psychiatry Hadamar, Hadamar, Germany; ^3^Department of Forensic Psychiatry and Psychotherapy II, Hospital of the Landschaftsverband Rheinland, Academic Teaching Hospital of the University of Cologne, Cologne, Germany; ^4^Medical Faculty, Institute for the History of Medicine and Medical Ethics, University Hospital, University of Cologne, Cologne, Germany; ^5^Chair of Social Philosophy and Ethics in Health Sciences, Department of Medicine, Faculty of Health Sciences, University Witten/Herdecke, Witten, Germany

**Keywords:** second language acquisition, schizophrenia, language, forensic psychiatry, language learning, longitudinal

## Abstract

**Background:** As a result of migration, an increasing number of patients in forensic psychiatric hospitals show poor skills in the national language, which can affect their treatment. Improving the second language (L2) of inpatients with schizophrenia may help to enable effective psychotherapy and thus reduce the risk of criminal recidivism and facilitate reintegration into society, for example because of a language-related higher degree of social functioning. For this purpose, a Hessian forensic psychiatric hospital established a ward specialized in L2 acquisition. The ward accommodates up to 21 patients with schizophrenia, who attend an L2 program consisting of 800–900 lessons within 1 year.

**Aims:** The study aimed to evaluate whether patients on the specialized ward (experimental group) achieve at least Common European Framework of Reference (CEFR) level A2 in the L2 program. Additionally, it examined whether language acquisition is better among participants in the experimental group than among those on regular wards (control group).

**Methods:** Achievements in the L2 were assessed by an L2 test 3 times: at the beginning of the program, after 6 months, and after 1 year. The impact of intelligence on achievements in L2 was evaluated using Raven's Standard Progressive Matrices.

**Results:** The experimental group showed significantly better improvement than the control group. Literacy was a significant predictor of improvement in the L2. The majority of the experimental group reached at least CEFR level A2 after 1 year.

**Conclusions:** High-intensity L2 programs are an effective way to improve the L2 of inpatients with schizophrenia in forensic psychiatric hospitals.

## Introduction

In accordance with the German legal system, offenders who commit serious crimes because of mental disorders are admitted to closed wards in forensic psychiatric hospitals. Similar to prisoners, forensic inpatients have to cope with deprivation of liberty, autonomy, and personal possessions (1). However, unlike incarceration, forensic psychiatric treatment aims to decrease the risk of recidivism by addressing the criminal risks associated with mental disorders (2).

The proportion of patients with a migration background in forensic psychiatry in Germany has risen since 2014 as a consequence of increased migration. Surveys in 2015 showed that 35.6% of forensic psychiatric inpatients in the federal state of Baden-Württemberg (Germany) had a migration background (3). A number of studies have been conducted on migrants in forensic psychiatry. The most frequent finding, is that migrants in forensic psychiatry are more likely to be diagnosed with schizophrenia and less likely to be diagnosed with personality disorders compared to patients without migration background. It was found for British, Canadian, Danish and German samples (4–7). This may reflect diagnosis biases related to poor language skills of migrants (8). However, Bulla et al. found that migrants from Southern Europe weren't more likely to be diagnosed with personality disorders compared to non-migrant patients in Baden-Württemberg. They speculate that German health professions may be familiar enough with the culture of South Europe which may decrease the risk of diagnosis biases (3). Moreover, a Canadian study found that migrants and non-migrants in forensic psychiatry didn't differ in sociodemographic variables such as age and education (5).

The high proportion of migrants in forensic psychiatry is a challenge for physicians, therapists, and nursing staff because linguistic and intercultural barriers make therapeutic work more difficult. In particular, poor language skills challenge the efficacy of psychotherapy and might lead to that forensic psychiatric treatment takes longer for non–native-speaking inpatients than for native-speaking inpatients.

One option to deal with poor language skills is to use an interpreter. Although this is a popular and apparently simple solution, it has several disadvantages in psychiatric settings. Interpreters must not be fellow inpatients or other non-professional persons (9) but have to be professional interpreters, so interpreting causes high additional costs and is logistically challenging. Furthermore, psychiatric interpretation requires good knowledge of mental disorders and psychotherapeutic techniques and close matching of the terminology of patients and psychotherapists. Such an approach might be feasible for frequently spoken languages, such as English, French or Spanish. However, for rarer ones, such as Tigrinya, these requirements are unlikely to be fulfilled. Furthermore, working in psychotherapeutic settings may cause a high level of emotional stress for interpreters (10). Consequently, a better long-term option may be to improve the second language (L2) of non–native-speaking forensic psychiatric inpatients.

Patients with schizophrenic disorders represent the largest diagnostic group (37%) in German forensic psychiatric hospitals (11). According to Dugan, a “wide variety of symptoms [of schizophrenia] are directly related to patients' ability to communicate” (12). However, recent reviews show that individuals with schizophrenic disorder are able to acquire new languages [e.g., (12, 13)]. For example, an Israeli study found that Russian migrants with and without schizophrenia showed similar patterns in spoken L2 after 5 years in Israel (14). The 2 groups differed only slightly in the syntax, lexis, and discourse markers evaluated in the study. The authors concluded that “despite the well-attested cognitive and social impairments in schizophrenia, second language learning proceeds rather normally” (14). Furthermore, psychotherapy in L2 may even have a positive impact on the treatment of schizophrenia (15, 16). Psychotherapy in patients' L2 is thought to trigger less emotional resonance than psychotherapy in their first language (L1). The reduced emotional resonance may be advantageous in cases where treatment can become emotionally overwhelming, such as in anxiety disorders.

Considering that almost all patients with a migration background in forensic-psychiatric hospitals in Germany want to remain in the country after their release, integration and networking in the host culture are essential components of rehabilitation programs. Thus, from a forensic-psychiatric perspective acquiring an L2 is not only important for making progress in psychotherapy, but also for successful reintegration into society. A recent review on the potential benefits of bilingualism for people with schizophrenia in Canada found that the employment rate was significantly higher in bilingual patients than in monolingual ones (13). The authors also assumed that learning an L2 may improve social functioning in patients with schizophrenia.

Besides the question whether patients with schizophrenia are in fact able to learn an L2, the extent of achievement isn't that clear. In Germany, the Common European Framework of Reference for Languages (CEFR) is used to assess language skills (17). CEFR levels A1 and A2 describe elementary language competence; people who reach this level can understand familiar and everyday words and use them in very simple sentences. At CEFR levels B1 and B2, individuals can talk about common and personal areas of interest and give brief explanations about them. CEFR levels C1 and C2 describe a competent use of language, and individuals with CEFR level C2 demonstrate near-native proficiency. Considering the importance of self-reflection in most psychotherapeutic approaches, CEFR-level B1 might be an appropriate minimum level for psychotherapy in an L2. To enable participants to reach CEFR level B1, L2 programs in Germany typically comprise 600 to 900 lessons (18).

In our study, we examined whether forensic inpatients with schizophrenia on the ward for language acquisition and integration were able to reach at least CEFR level A2 or even B1 in German within 1 year. We compared the progress of this group of patients in 1 year with that of patients at other forensic psychiatric hospitals who participated in regular treatment on wards that were not specialized in language acquisition.

## Methods

### Procedure and Participants

The experimental group (EG) comprised patients from the forensic psychiatric ward specialized in language acquisition and integration at the Vitos Clinic for Forensisc Psychiatry in Hadamar. Patients of the ward were male, have committed a crime as a result of a schizophrenic disorder and have little or no knowledge of German. The ward was established to provide more targeted support to meet the specific needs of this patient population. It accommodates up to 21 inpatients who are first-generation migrants. The focus of the work on the ward is to teach German in everyday clinical practice through both intensive instruction and practical applications. Inpatients received 20 German lessons per week. For literate inpatients, the entire L2 program comprised 800 lessons, and for illiterate inpatients, 900 lessons, including a preceding unit of literacy instruction. The L2 program was separated into 4 successive parts that progressed from CEFR level A1.1 to level A2.2. The curriculum of the Federal Office for Migration and Refugees in Germany served as the basis for the L2 programs (18, 19). All teachers are certified by the Federal Office as teachers of German as a Second Language.

To recruit patients for the control group (CG), 7 German forensic psychiatric hospitals were contacted by email. They were asked if they treat patients who had the following inclusion criteria: First generation migrants with little or no knowledge of German who speak an L1 equivalent to that of a patient in the EG. Six forensic psychiatric hospitals agreed to participate in the study. Although these patients on regular wards had daily contact with German-speaking fellow inpatients, unlike the inpatients in Hadamar, they were only given German lessons if they requested them, and the lessons were conducted less often than in Hadamar.

Patients in EG and CG were first-generation migrants. All patients had a schizophrenic disorder (F2 according to ICD-10 criteria), which was diagnosed by experienced clinicians. Additionally, in EG, 10 patients had a disorder due to psychoactive substance use (F10-F19), 1 patient had a neurotic disorder (F40-F48), 1 patient had a intellectual disability (F70-F79) and 1 patient had a mental disorder with onset in childhood or adolescence (F90-F98). In CG, 4 patients had a disorder due to psychoactive substance use (F10-F19) and 1 patient a personality disorder (F60-F69). Participants were informed about the procedure and purpose of the study, signed informed consent, participated voluntarily and received no compensation.

Data were collected in the years 2017–2021 at 3 times: baseline (T1) and after 6 months (T2) and 1 year (T3). At T1, we collected sociodemographic data (age, education, and information on literacy) and tested participants' intelligence with Raven's Standard Progressive Matrices (RPM). At T2, we assessed the psychological distress of the sample using the Brief Symptom Checklist (BSCL). At T1, T2, and T3, we assessed all participants' German language skills with the L2 test *Pluspunkt Deutsch*. In the EG, the L2-test was conducted as part of the L2 program by the language teachers, in the CG they were conducted by a research assistant. Participants in the EG received periodic feedback about their progress in German within the context of the L2 program, and those in the CG received feedback about their results in the L2 test upon request.

The number of participants reduced between T1 and T3. At T1, we recruited 28 participants in the EG and 30 in the CG. At T2, 26 participants of the EG (92.86%) and 21 participants of the CG (70.00%) were tested and at T3, 18 participants of the EG (64.29%) and 18 of the CG (60.00%) remained. Most of the patients who could no longer be tested had left the clinic/ward during the course of the survey. Other patients refused to continue attending the language course and still others refused to take the language test.

### Measures

#### L2 Test—*Pluspunkt Deutsch*

*Pluspunkt Deutsch* is an L2 test that assesses the current CEFR level (20, 21). It consists of 40 multiple choice items with different tasks. The tasks consist of word order and word completion tasks, in which participants have to choose the right word or phrase for the respective task, and decision tasks, in which participants have to decide whether a statement is true or false. The test has 3 successive subtests, A1, A2, and B1. In our study, we assessed participants with tests A1 and A2. Correct answers were summed to obtain a total score/CEFR level (Table 1). For statistical analysis, we merged the scores of both tests into a single scale ranging from 0 (A1.1) to 4 (B1.1).

**Table 1:** Scoring of the L2 tests.

**Test A1**		**Test A2**	
**Score**	**CEFR level**	**Score**	**CEFR level**
0–17	A1.1	0-17	A2.1
18–33	A1.2	18-33	A2.2
33+	A2.1	33+	B1.1

#### Raven's Standard Progressive Matrices

RPM (22) is a widely used test to estimate fluid intelligence. It was developed to provide a non-verbal measure of intelligence and consists of 60 items that gradually increase in difficulty (23). The task is to select the figure from 6 to 8 options that fits the pattern of the current item. The number of correct answers is summed to give a total score and is then transformed to a standardized T value that compares the participant's individual total score with those of people in the same age group. For our purpose and because of the cognitive limitations of inpatients with schizophrenia, we used a short form of the RPM with 32 Rasch homogeneous items (24). The short form was developed for the Vienna Test System and has been standardized by age in a sample with *n* = 299 and has a reliability of 0.91.

Despite the use of a short form of RPM, some participants answered an extremely low number of items correctly, so we were unable to transform the raw scores of these participants into standardized T-values. The low scores may reflect cognitive, educational, or cultural limitations in the use of the RPM (see section Limitations). Therefore, we decided to exclude these results from our examination of the impact of intelligence on language acquisition. Therefore, the reported results should be interpreted with caution.

#### Brief Symptom Checklist

The BSCL (11) is a self-assessment instrument for measuring psychological distress by asking for psychiatric symptoms (25). It was originally published as Brief Symptom Inventory (26). It consists of 53 items (Cronbachs Alpha for the Total Score = 0.97) which ask for Hostility, Anxiety, Depression, Paranoid Ideation, Phobic anxiety, Psychoticism, Somatization, Interpersonal Sensitivity and Obsession-Compulsion. For our purpose it was translated into the L1 of the participants.

### Data Analyses

Data Analyses was conducted using IBM SPSS Statistics for Windows, Version 27 (27).

Studies investigating L2 skills usually suffer from low sample sizes (12). This is a quite problematic issue for statistical analysis especially when sample size decrease within longitudinal studies as a result of dropout. Therefore, imputation using last observation carried forward (LOCF) was used to address the dropout in the sample for analyzing the language acquisition of both EG and CG within 1 year (=LOCF-Model). LOCF is a method to handle missing data which uses the last observed individual value of a measure to impute the values of the further observations. For example, for a participant who dropped out after T1, the observed CEFR level at T1 was imputed as CEFR level at T2 and T3. A disadvantage of LOCF is that it may under- or overestimate effects of interventions (28). In our study, LOCF is more likely leading to decrease mean values at later observations because it assumes that the CEFR levels of the dropout group didn't increased after dropout of the study. Therefore, we also computed a model which excludes participants of the dropout group or rather only includes participants who were examined at all observations (=Exclusion-Model). This model estimates the effects of the intervention for participants who finished the whole L2 program in EG. Both models were analyzed using mixed between-within ANOVAs.

Dropout of participants may occurred as a result of important variable such as intelligence (RPM-score), literacy or L2 skills. Therefore, we included a dropout analysis using student's *t*-tests, *U*-tests and chi-square tests.

## Results

### Descriptive Statistics

Table 2 shows the descriptive data of the sample.

**Table 2:** Descriptive statistics of the participants in the experimental (EG) and control group (CG).

	**EG**	**CG**	**Statistics**
Age	*M* = 31.71, *SD* = 8.60	*M* = 30.33, *SD* = 7.18	*t*_(56)_ = −0.67, *p* = 0.508
First language	Arabic: 5 (17.9%) Tigrinya: 7 (25.0%) Somali: 3 (10.7%) Portuguese: 1 (3.6%) Polish: 1 (3.6%) Farsi: 1 (3.6%) Dutch: 2 (7.1%) Serbian: 2 (7.1%) Igbo: 1 (3.6%) Hungarian: 1 (3.6%) Spain: 1 (3.6%) Romanian: 1 (3.6%) Turkish: 1 (3.6%) Kurdish: 1 (3.6%)	Arabic: 10 (33.3%) Tigrinya: 3 (10.0%) Somali: 3 (10.0%) Portuguese: 1 (3.3%) Polish: 2 (6.7%) Farsi: 2 (6.7%) English: 3 (10.0%) French: 1 (3.3%) Albanian: 1 (3.3%) Edu: 1 (3.3%) Pashto: 1 (3.3%) Ashanti 2 (6.7%)	*χ^2^*_(19)_ = 22.89, *p* = 0.242
Education			*U* = 344.00, *p* = 0.480
No graduation	14 (56.0%)	17 (68.0%)	
Graduated after 9 or 10 years of school	7 (28.0%)	4 (16.0%)	
General qualification for university entrance	4 (16.0%)	4 (16.0%)	
Psychological distress (BSCL)	*M* = 23.16, *SD* = 30.30	*M* = 41.31, *SD* = 37.89	*t*_(30)_ = 1.51, *p* = 0.143

*Mental State: A higher score indicates a higher level of psychological distress. BSCL, Brief Symptom Checklist*.

Table 3 shows the individual results of the EG and CG in the L2 test at baseline (T1) and after 1 year (T3).

**Table 3:** Results of participants in the experimental group (EG) and control group (CG) at baseline (T1) and after 1 year (T3).

		**EG**		**CG**	
	**CEFR level**	**Literate**	**Illiterate**	**Literate**	**Illiterate**
T1	A1.1	14 (77.8%)	9 (90.0%)	14 (66.7%)	8 (88.9%)
	A1.2	4 (22.2%)	1 (10.0%)	5 (23.8%)	1 (11.1%)
	A2.1	0 (0.0%)	0 (0.0%)	2 (9.5%)	0 (0.0%)
T3	A1.1	0 (0.0%)	2 (33.3%)	7 (46.7%)	3 (100.0%)
	A1.2	0 (0.0%)	3 (50.0%)	1 (6.7%)	0 (0.0%)
	A2.1	2 (16.7%)	1 (16.7%)	3 (20.0%)	0 (0.0%)
	A2.2	5 (41.7%)	0 (0.0%)	1 (6.7%)	0 (0.0%)
	B1.1	5 (41.7%)	0 (0.0%)	3 (20.0%)	0 (0.0%)

*CEFR, Common European Framework of Reference for Languages*.

### Dropout Analysis

To examine whether patients who dropped out of the study during the course differed from those who continued to participate, a drop-out analysis was performed. As can be seen in Table 4, the two groups did not differ in any of the variables studied.

**Table 4:** Mean values (*M*), median (*Md*) and standard deviations (*SD*) for the examined variables in the dropout analysis of the experimental group (EG) and control group (CG).

	**Dropout**	**Non-dropout**	**Statistics**
**CEFR level at T1**			
EG	*n* = 10, * M* = 0.10, *SD* = 0.32	*n* = 18, *M* = 0.22, *SD* = 0.43	*t*_(26)_ = 0.79, *p* = 0.437
CG	*n* = 12, *M* = 0.17, *SD* = 0.39	*n* = 18, *M* = 0.44, *SD* = 0.70	*t*_(28)_ = 1.24, *p* = 0.225
**CEFR level at T2**			
EG	*n* = 9, *M* = 1.00, *SD* = 1.22	*n* = 17, *M* = 0.94, *SD* = 0.90	*t*_(24)_ = −0.14, *p* = 0.890
CG	*n* = 4, *M* = 0.50, *SD* = 0.58	*n* = 17, *M* = 0.71, *SD* = 0.85	*t*_(19)_ = 0.46, *p* = 0.653
**Illiterate participants**			
EG	*n* = 4 (40.0%)	*n* = 6 (33.3%)	χ^2^(1) = 0.124, *p* = 0.724
CG	*n* = 6 (50.0%)	*n* = 3 (16.7%)	χ^2^(1) = 3.810, *p* = 0.051
**RPM-score (intelligence)**			
EG	*n* = 4, *M* = 78.25, *SD* = 21.30	*n* = 14, *M* = 75.43, *SD* = 12.83	*t*_(16)_ = −0.22, *p* = 0.931
CG	*n* = 8, *M* = 80.59, *SD* = 18.50	*n* = 13, *M* = 74.96, *SD* = 21.25	*t*_(19)_ = −0.63, *p* = 0.537
**Age**			
EG	*n* = 10, *M* = 29.00, *SD* = 8.19	*n* = 18, *M* = 33.22, *SD* = 8.67	*t*_(26)_ = 1.26, *p* = 0.220
CG	*n* = 12, *M* = 32.08, *SD* = 4.48	*n* = 18, *M* = 29.17, *SD* = 8.45	*t*_(28)_ = −1.09, *p* = 0.283
**Education**			
EG	*n* = 10*, Md* = 0.00	*n* = 15*, Md* = 0.00	*U* = 73.00, *p* = 0.901
CG	*n = 8, Md* = 0.00	*n = 17, Md* = 0.00	*U* = 65.00, *p* = 0.832
**Psychological distress (BSCL)**			
EG	*n* = 5, *M* = 14.80, *SD* = 28.70	*n* = 14, *M* = 26.14, *SD* = 31.33	*t*_(17)_ = −0.71, *p* = 0.488
CG	*n* = 4, *M* = 54.00, *SD* = 30.08	*n* = 9, *M* = 35.67, *SD* = 41.08	*t*_(11)_ = −0.80, *p* = 0.444

*n = number of participants; Education: 0 = No Graduation, 1 = Graduated after 9 or 10 years of school, 2 = General qualification for university entrance; Mental State: A higher score indicates a higher level of psychological distress. BSCL, Brief Symptom Checklist; CEFR levels: 0 = A1.1, 1 = A1.2, 2 = A2.1, 3 = A2.2, 4 = B1.1*.

### Analysis of the L2 Skills in EG and CG Within 1 Year

Table 5 shows an overview of means and standard deviations of the examined variables in the two groups.

**Table 5:** Overview of the variables examined in the experimental (EG) and control group (CG).

	**Observed**	**Exclusion-model**	**LOCF-model**
**Number of participants**			
EG	T1: *n* = 28 T2: *n* = 26 T3: *n* = 18	*n* = 17	*n* = 28
CG	T1: *n* = 30 T2: *n* = 21 T3: *n* = 18	*n* = 17	*n* = 30
**CEFR level at T1**			
EG	*M* = 0.18, *SD* = 0.39	*M* = 0.24, *SD* = 0.44	*M* = 0.18, *SD* = 0.39
CG	*M* = 0.33, *SD* = 0.61	*M* = 0.47, *SD* = 0.72	*M* = 0.33, *SD* = 0.61
**CEFR level at T2**			
EG	*M* = 0.96, *SD* = 1.00	*M* = 0.94, *SD* = 0.90	*M* = 0.89, *SD* = 0.99
CG	*M* = 0.67, *SD* = 0.80	*M* = 0.71, *SD* = 0.85	*M* = 0.50, *SD* = 0.73
**CEFR level at T3**			
EG	*M* = 2.44, *SD* = 1.38	*M* = 2.59, *SD* = 1.27	*M* = 1.89, *SD* = 1.50
CG	*M* = 1.22, *SD* = 1.59	*M* = 1.29, *SD* = 1.61	*M* = 0.83, *SD* = 1.34
**Illiterate participants**			
EG	*n* = 10 (35.71%)		
CG	*n* = 9 (30.00%)		
**German lessons per week**			
EG	*M* = 20, *SD* = 0.00		
CG	*M* = 2.24, *SD* = 1.22		
**Intelligence (RPM)**			
EG	*M* = 76.83, *SD* = 14.37		
CG	*M* = 20, *SD* = 0.00		
**Change in CEFR level over 1 year**			
EG	*M* = 2.22*, SD* = 1.26		
CG	*M* = 0.78, *SD* = 1.00		

*M, Mean; SD, Standard deviation; CEFR, Common European Framework of Reference for Languages; n, number of participants; RPM, Raven's Standard Progressive Matrices; T1 = baseline, T2 = half a year, T3 = 1 year.*

*CEFR levels: 0 = A1.1, 1 = A1.2, 2 = A2.1, 3 = A2.2, 4 = B1.1.*

*Observed = values as observed for participants at T1, T2 and T3 without imputation or exclusion.*

*Exclusion-Model: Excludes participants who showed missing data at any observation.*

*LOCF-Model: Imputation Model which includes the full sample. For participants who dropped out and therefore showed missing data, the last observed CEFR level was used for imputation (=last observation carried forward method)*.

Table 6 shows the results of the between-within linear models which were computed to analyze achievements in the CEFR level within 1 year in EG and CG.

**Table 6:** Summary of the exclusion-model and the LOCF-model for achievements in the German CEFR level in the experimental group and control group.

	**Exclusion-Model (** ***n*** **=** **34)**			**LOCF-Model (** ***n*** **=** **58)**		
	***F***	***df***	***r***	***F***	***df***	***r***
Time	30.43^***^	1.80, 54.04	0.62	28.20^***^	1.75, 94.28	0.48
Group	2.47	1, 30	0.28	4.51^*^	1, 54	0.28
Literacy	11.81^**^	1, 30	0.53	16.79^***^	1, 54	0.48
Time ^*^ Group	10.71^***^	1.80, 54.04	0.41	11.27^***^	1.75, 94.28	0.33
Time ^*^ Literacy	10.42^***^	1.80, 54.04	0.40	14.68^***^	1.75, 94.28	0.37
Time ^*^ Group ^*^ Literacy	1.00	1.80, 54.04	0.18	1.60	1.75, 94.28	0.13
Group ^*^ Literacy	0.11	1, 30	0.06	0.29	1, 54	0.07

*Dependent variable = Common European Framework of Reference for Languages (CEFR) level;*

* *p < 0.05,*

** *p < 0.01,*

*** *p < 0.001.*

*Exclusion-Model: Excludes participants who showed missing data at any observation.*

*LOCF-Model: Imputation Model which includes the full sample. For participants who dropped out and therefore showed missing data, the last observed CEFR level was used for imputation (= last observation carried forward method)*.

Both models showed a significant main effect of time and literacy. The main effect group was significant in the LOCF-Model, however, non-significant in the Exclusion-Model. Both models showed a significant interaction between time and group, that is, participants in the experimental group achieved a significantly higher CEFR-level within 1 year than participants in the control group (as can be seen in Table 5). In addition, both models showed a significant interaction between time and literacy meaning that literate patients achieved a higher level of language proficiency within 1 year than non-literate participants (as can be seen in Table 3).

In a correlation analysis, we investigated the relationship between RPM-score and progress in language acquisition. The correlation between the RPM-score and the mean achievement in L2 over 1 year was not significant *r* = 0.36, *p* = 0.066. Further analysis showed that the 2 groups did not differ in terms of mean RPM-score, mean difference = −0.31; BCa 95% CI (−10.69, 11.49) and *t*_(37)_ = −0.124; *p* = 0.958.

## Discussion

As stated in the Introduction, previous research repeatedly found that patients with schizophrenia are able to learn an L2 (12, 13). The results of the present study confirm these findings. We computed two models, an Exclusion Model and a LOCF-Model which both agree in their main findings. The significant effect of time reflects that participants in both, EG and CG improved their L2 skills (CEFR level) within 1 year. However, the significant interaction between time and group indicates that improvements in L2-skills were stronger for participants in the EG. This suggests that the L2 program in the ward for language acquisition and integration was more effective in improving German than usual language acquisition efforts in forensic psychiatry. The Exclusion-model shows the effects of L2 programs for participants who finished the whole program. Effect sizes were moderate (*r* = 0.40) to large (*r* = 0.60) with respect to effect sizes which were typically found in L2 research (29).

However, the exclusion of the dropout group may lead to an overestimation of the language acquisition especially in the EG. The LOCF-Model which also includes the participants of the dropout group shows smaller effect sizes (0.30 ≤ *r* ≤ 0.50) than the Exclusion model. They may be more realistic considering that dropout of participants in L2 programs is quiet normal even in the general population (30). However, the imputation of L2 scores in the dropout group using LOCF may also lead to a biased estimation of the language acquisition. The majority of participants showed CEFR level A1.1 or A1.2 at the last observation which was used for the imputation of the following observations. Therefore, the LOCF-Models assumes that participants of the dropout group remained at an elementary level of German (17). This may underestimate the language acquisition, taking in account that in both models the main effect of time was significant which suggests that the L2 skills would have improved over time. Taking the overestimation of the language acquisition in the Exclusion-Model and the underestimation in the LOCF-Model together, this could suggest that the true effect sizes may fall between the estimates of the two models.

However, the important role of literacy should be noted. The significant interaction between time and literacy reflects that L2-skills in literate participants increased more compared to illiterate ones. In addition, the non-significant interaction between group and literacy shows that literacy wasn't more meaningful in neither the EG nor the CG. Moreover, there were a non-significant interaction between time, group and literacy. This means that literacy neither in the EG nor in the CG was more related to the observed improvements in the L2 skills within 1 year. Taken together, illiterate participants were disadvantaged with respect to general improvements in L2-skills. In addition, the high intense L2 program wasn't as effective as for literate participants in the EG.

The important role of intelligence for school achievement has been repeatedly demonstrated. A recent meta-analysis found an overall mean correlation between intelligence and school achievement of 0.41 ≤ *r* ≤ 0.48 (31). For non-verbal measures such as RPM, the meta-analysis typically found a lower mean correlation of 0.34 ≤ *r* ≤ 0.43. The effect size of the barley non-significant correlation between RPM-scores and the mean achievement of *r* = 0.36 found in the present study corresponds with the above findings. However, two of our findings are noteworthy. First, the participants in the EG and CG did not differ significantly in RPM-score. Therefore, the EG's better achievements in learning German can be considered as a result of explicit language acquisition. Second, the observed mean in RPM-score of participants in both the EG and CG was below average; however, participants in the EG were nevertheless able to successfully increase their CEFR level.

Another question addressed by this study was how well patients with schizophrenia learn an L2. In Germany, 91.8% of people in the general population who finish general L2 programs reach CEFR level A2 or B1 (30). The L2 achievements of the literate participants in the EG were comparable to those of the general population in general L2 programs. However, the achievements of the illiterate participants were clearly worse than those of participants in literacy programs in the general population, where 59.3% reached at least CEFR level A2 (30). Thus, as long as inpatient migrants with schizophrenia are literate, they can be considered to be as able as migrants without schizophrenia to successfully participate in L2 programs. To achieve better proficiency in German, illiterate inpatients might need additional support, such as a higher number of lessons.

In sum, although participants in the EG successfully improved their CEFR level, the majority of participants in the CG did not. Language acquisition support for participants in the EG provided good conditions for them to improve their L2. One important condition might be the number of lessons per week. Participants in the EG received 20 German lessons per week, which was almost 10 times more than the mean number received by the CG. Moreover, participants in the EG were taught on 5 days a week. Studies have repeatedly found that schizophrenia is associated with deficits in working memory (32), which plays an important role in encoding new information. Thus, participants in the CG may have failed to consolidate newly learned words or phrases into long-term memory because language lessons were not frequent enough. Unlike the EG, the CG had the opportunity to socialize with fellow patients whose native language is German. Thus, they had the opportunity to practice German language skills in everyday conversation. However, the results of the present study do not indicate that these opportunities significantly support language acquisition. Another reason for the good performance of the EG may be that group instruction were more advantageous than individual instruction. In group instruction, teachers may be less able to delay successive language units due to a slower learning rate of an individual patient, for example, to repeat the last unit, than in individual instruction. This may lead to a faster progress in the L2 program and therefore a shorter total learning time. Furthermore, the EG were in a motivational environment that supported participants in improving their L2. Motivation is an important predictor of language acquisition (31). In the EG, language acquisition was a mandatory goal of both inpatients and staff, so inpatients were constantly encouraged to practice the L2.

## Limitations

The sample size of the examined sample is low. This might be quite normal for studies investigating L2 acquisition in patients with schizophrenia (12). Nevertheless, it may have a negative effect on statistical computations. For example, we were unable to match the EG and CG for age and L1 because of the decrease in sample size over time. In addition, important subgroups such as for intelligence, educational background or different L1 could not be analyzed. Further research is needed to investigate the impact of those predictors.

As reported above, some participants were illiterate. Therefore, the L2 tests were read to these participants. In contrast, the literate participants worked through the L2 test independently. Thus, in literate participants the L2 tests were related to reading comprehension, but in illiterate participants they were rather related to listening comprehension.

Language skills were tested with 2 different L2 tests that were associated with particular CEFR levels. Therefore, the tests showed both an upper and a lower limit. We only examined whether participants reached CEFR level B1.1, but some participants may have exceeded this level.

The RPM is a measure for the non-verbal assessment of intelligence which can handle validity problems caused by lacking language skills. However, non-verbal measures such as RPM also show problems in validity caused by culture differences (33). For example, while European participants are usually used to figurative tasks in school, this cannot be taken for granted for participants of countries with poorly developed educational system. As stated before in the method section some participants showed an extremely low number of correct items in RPM. This may reflect educational and cultural limitations in the use of the RPM.

The BSCL was translated into the L1 of the participants. This may impact the validity of the measure. In addition, due to illiteracy some participants couldn't be asked. That's why the results of the BSCL should be interpreted with caution.

## Conclusions and Practical Implications

Our results on the effects of the language program, which consisted of frequent lessons, were encouraging, and the participants were able to successfully improve their language skills. Therefore, L2 acquisition programs may be a good option for addressing language-related problems in the treatment of forensic inpatients.

To achieve good results both staff and inpatients must be motivated to engage in inpatients' L2 acquisition. Language acquisition is a time-demanding task that may not lead to measurable improvements for several weeks, despite daily lessons. If improving L2 skills is voluntary, other demands of forensic treatment may interfere with the commitment of patients and staff to improve L2 skills because they may appear to be more important than language acquisition.

Language skills are a general resource and affect several domains. For example, both inpatients in forensic psychiatry and people in long-term imprisonment commonly worry about becoming a victim of criminal behavior (34, 35). Poor relationships or a lack of relationships with other prisoners was found to be associated with a fear of crime in migrants in long-term imprisonment (36). Further studies may investigate whether L2 programs help to improve relationships with fellow patients and therefore decrease the fear of crime. Providing good living conditions is important for patients with frequent, long-term stays in forensic psychiatry (37). Furthermore, as stated in the Introduction, reintegration into society is the main goal of forensic psychiatry. However, the secondary benefits of language acquisition, such as a higher level of social functioning in patients with schizophrenia, should not be underestimated (13). In addition, the impact of L2 programs on length of stay in forensic psychiatry or recidivism after discharge should be investigated.

## Data Availability Statement

The raw data supporting the conclusions of this article will be made available by the authors, without undue reservation.

## Ethics Statement

The studies involving human participants were reviewed and approved by Ethics review committee of the State Chamber of Physicians of Hesse. The patients/participants provided their written informed consent to participate in this study.

## Author Contributions

MD and NR designed the study. ML and MD were responsible for administration of data collection. ML and LT conducted the literature research. ML wrote the first draft of the paper. ML and MB conducted the statistical analysis. JS supervised the statistical analysis and writing process. JS, MD, CP-S, and LT revised the manuscript. All authors read and approved the final version of the manuscript.

## Conflict of Interest

The authors declare that the research was conducted in the absence of any commercial or financial relationships that could be construed as a potential conflict of interest.

## Publisher's Note

All claims expressed in this article are solely those of the authors and do not necessarily represent those of their affiliated organizations, or those of the publisher, the editors and the reviewers. Any product that may be evaluated in this article, or claim that may be made by its manufacturer, is not guaranteed or endorsed by the publisher.
